# Analysis of the Mediating Effect of Risk Perception on the Relationship Between Time Perception and Mental Health of College Students During the COVID-19 Epidemic

**DOI:** 10.3389/fpsyt.2021.749379

**Published:** 2021-11-10

**Authors:** Hua Cao, Hui Wen Zhang, Ling Yang, Ling Li, Jun Zhi Wang, Bakht Zada, Min Xiao Li, Wei Jian Liu, Ting Hong Su, Yu Zhao

**Affiliations:** School of Psychology, Northwest Normal University, Lanzhou, China

**Keywords:** COVID-19, college students, time perception, mental health, risk perception

## Abstract

**Background:** COVID-19 has had a wide impact on the mental health of college students. This study aims to explore the relationship between time perception, risk perception, and the mental health of college students during COVID-19 through a questionnaire survey.

**Subjects:** One thousand two hundred and eighteen college students, 449 male and 769 female, who studied online during the COVID-19 epidemic were selected.

**Methods:** Time Perception Scale, Risk Perception Scale, and SCL-90 were used to investigate the relationship using correlation analysis.

**Results:** During the COVID-19 period, mental health problems of college students were widespread, and 65.93% of college students reported moderate to severe mental health problems. The correlation analysis showed that risk perception, time perception, and the mental health of college students were significantly related. Risk perception played a partial mediating role between present enjoyment and mental health, and risk perception played a partial mediating role between future time perception and mental health.

**Conclusion:** In the case of sudden public crises, we should pay close attention to the mental health of college students, adjust their attitude toward the present and the future, and pay attention to their perception of risk so as to improve their mental health level under crisis.

## Background

Novel coronavirus (COVID-19 for short) has spread all over the world. Because of its fast transmission speed, wide transmission range, strong infectivity, and lack of targeted treatment, it poses a significant threat to people's physical and mental health (1). Sudden public crisis events can easily lead to psychological reactions such as tension, anxiety, and even panic, which lead to psychological barriers such as stress and depression (2). As a special group in the epidemic, college students are more vulnerable to the impact, because they face double pressure from home isolation and complete online learning, and suffer greater impact and serious mental health problems. Studies have shown that 20–40% of Chinese adolescents were prone to severe psychological issues, especially anxiety and post-traumatic stress disorder (PTSD) symptoms, during the COVID-19 pandemic (3). However, in a public health crisis, the mental health needs of college students may be ignored, and due to the lack of control over their own environment, they tend to bear more significant mental health pressure (4). For college students, they left school during the COVID-19 pandemic, which has had a significant impact on their physical and mental health. Some college students even experienced poor sleep quality, loss of appetite, increased sedentary behavior, and loss of cardiometabolic health (5). Therefore, it is of great practical and theoretical significance to study the impact of this sudden public crisis on college student's mental health and how to help college students better cope with the crisis and maintain their mental health.

Previous studies have found that individuals' time orientation affects their behavior in major public crisis events. The prospect of the future helps people to formulate effective protection measures to avoid being affected by high-risk activities (6). The perception of time and our perception of the future may affect our physical and mental health. Time perception is an important reflection of individual tendencies, which has a significant impact on individual emotions and behaviors. Research shows that time perception is strongly associated with anxiety, depression, self-esteem, happiness, and life satisfaction (7, 8). Moreover, as a personality trait, insight has an important impact on individual emotions and behaviors. Previous studies have found that time insight is closely related to individual health behaviors (9). Studies have shown that altered perceptions of time and its passage are common experiences of trauma (10, 11). As a basic dimension of constructing psychological time, Zimbardo and Boyd define time insight as an unconscious process, which defines the sequence, relevance, and meaning of these events by dividing the continuous stream of personal and social experience into different time categories (12). Zimbardo and Boyd divided time perception into five dimensions: past negative, past positive, present fatalism, present hedonism, and future. All of these were significantly correlated with individual anxiety level (12). Past negative and present fatalism were significantly positively correlated with anxiety disorder. Future time orientation was negatively correlated with anxiety disorder. Sudden major events may change an individual's evaluation of the present and future, which may lead to a high degree of psychological pain and affect the level of individual mental health. Individuals with a positive time attitude have a positive and optimistic view of the past, present, or future, and are more likely to make positive expectations when facing unknown dangerous events, thus reducing psychological anxiety and anxiety caused by dangerous situations and putting individuals in a lower state of anxiety (10). Moreover, studies have found that time perception is closely related to individual health, and time perception can affect individual psychological experience (9). The stronger an individual's tendency toward future time, the better they can adapt to school life (13). When an individual pays too much attention to the past and is content with the present situation, they will commit more crimes. In contrast, an individual who pays more attention to the present thinks less about the future. This shows that when an individual is confident in the future and has a more positive attitude toward the future, it is easier for them to face difficult situations more positively. Conversely, when individuals are immersed in the past and content with the status quo, they will adopt more negative ways to solve stress events.

During the outbreak period, individuals were faced with the risk of infection. The perceived degree of risk and the uncertainty and uncontrollability of risk will increase the psychological pressure and negative emotions of individuals, seriously affecting the mental health of the public (14). Risk perception refers to an individual's subjective judgment for the risk based on the objective crisis. In the face of the COVID-19 pandemic, the uncertain environment will threaten individuals, and they will subjectively judge the possibility of their infection; this is the core of risk perception (15). Studies have also shown that high risk perceptions can put people in a state of depression and anxiety. The occurrence of risk events will create a stressful environment for people and generate negative emotions such as anxiety and tension, resulting in certain mental health problems (16). Previous studies on risk perception of events such as floods, earthquakes, and terrorist attacks have shown that perception of risk events has an impact on psychological responses (17). The high uncertainty and low sense of control of risk events will induce strong emotions such as worry. High familiarity and high sense of control will reduce emotional and other aspects of the response accordingly. Taking SARS as the object, researchers studied the public's risk perception in epidemic infectious disease crisis events and found that the public's risk perception of information related to public health crisis events significantly affected their mental health (14). Griva et al. found that future time perception was associated with risk perception, and women who considered the future consequences of their actions were more likely to consider health-related risks. As an emotional component of time, time attitude is also associated with risk perception. The higher an individual perceives the risk of an event, the greater the degree of anxiety and panic brought about by the event, which leads to the improvement of anxiety level and lower mental health level (3). To sum up, there may be a relationship between risk perception, time perception insight, and the mental health of college students. Based on this in this study, we propose the following hypotheses:

Hypothesis 1: During the COVID-19 pandemic, future time perception is negatively correlated with the mental health of college students.

Hypothesis 2: There is a negative correlation between the dimension of present hedonism and the mental health of college students during the COVID-19 pandemic.

Hypothesis 3: Risk perception plays a partially mediating role between future time perspective, present hedonism, and mental health during the COVID-19 pandemic.

## Methods

### Participants

In this study, questionnaire star was used to issue questionnaires. Questionnaires were issued in Gansu, Sichuan, Guizhou, Henan, Hebei, Shaanxi, Chongqing, and 12 other provinces (not including Hubei, given that Hubei province is in the midst of the outbreak, and the psychological health problems of college students' mental, time orientation, and risk perception may be extremely influenced. Therefore, the data collection process mainly took place for HuBeioutside college students). A total of 1,335 questionnaires were collected, including 1,218 valid ones. The basic information of respondents is shown in Table 1. The age of the subjects was 20.32 ± 1.52 years.

**Table 1:** Basic information of the respondents.

		***N***	**%**
Gender	Male	449	36.9
	Female	769	63.1
National	Han nationality	1,014	83.3
	Ethnic minorities	204	16.7
Family Residence	Rural	900	73.9
	Cities and towns	318	26.1
Grade	Freshman	637	52.3
	Sophomore	300	24.6
	Junior	154	12.6
	Senior	127	10.4

### Time Perception Scale

The revised Chinese version of the Zimbardo Time Insight Short-Version Questionnaire (ZTPI) was adopted. The questionnaire consists of 20 items and is divided into five dimensions: Past Negative PN, Past Positive PP, Present Hedonistic Ph, Present Fatalistic PF, and Future F. This questionnaire is a 5-point Likert scale. According to their own situation, the subjects choose from 1 (significantly disagree) to 5 (significantly agree). The Cronbach's α coefficient of the five dimensions ranged from 0.54 to 0.68, and the Cronbach's α coefficient of the whole scale was 0.66 (18).

### SCL-90

SCL-90 revised by Zhengyu Wang was adopted, and the scale contained 10 factors. The full scale consisted of 90 items, each of which was rated on a 5-point scale (1–5), with 1 = none and 5 = severe. The statistical indicators of the scale were the total mean score and factor score. In this study, the total coefficient of A of the scale was 0.978, and the coefficient of A of each factor ranged from 0.772 to 0.921 (19).

### Risk Perception Scale

The risk perception questionnaire was based on Slovic's risk perception model. Two indicators, familiarity and control, were used to investigate the public's risk perceptions of six risk sources of COVID-19 (the etiology, transmissibility and infectivity of COVID-19, cure rate, preventive measures, effects on the body after treatment, and whether the disease is infective after treatment). It was measured using the Likert 5-point scale. Cronbach's α coefficient of the total amount table is 0.793 (20).

### Statistical Analysis

SPSS22.0 and the PROCESS plug-in developed by Hayes were used for analysis. In this study, 5,000 samples were put back from the original samples to estimate the 95% confidence interval of the mediating effect. In addition, model 4 with 76 typical models provided by Hayes was selected for analysis.

## Results

Table 2 provides mental health of college students' characteristics. In the present sample, 64.7% of college students have moderate to severe mental health problems, and there is no significant difference in gender, age, and grade (*P* > 0.05). This means that during the COVID-19 pandemic, college students generally have mental health problems.

**Table 2:** Mental health of college students during the COVID-19 pandemic.

**Level**	***N***	**%**
160–200	429	35.2
200–250	389	31.9
>250	400	32.8

*Level < 160, mental health level is normal, Level in 160–200, mild mental health problems, Level in 200–250, moderate mental health problems, Level > 250, severe mental health problems*.

Table 3 provides means, standard deviations, and Pearson correlations of the main study variables. Here, College Students' Mental Health (MH) is associated with a lower familiarity dimension and controllability dimension of Risk Perception (RP), with more negative past dimension, present hedonism dimension, and fatalism dimension of Time Perspective (TP), but more future time concept dimension of TP. On the other hand, the familiarity dimension of RP is associated with a more positive past dimension, present hedonism dimension, and future time dimension of TP. In contrast, the controllability dimension of RP is associated with a more positive past dimension, present hedonism dimension, and future time dimension of TP.

**Table 3:** Correlation analysis between risk perception and college students' mental health and time insight during the COVID-19 epidemic.

	**1**	**2**	**3**	**4**	**5**	**6**	**7**	**8**
1. MH	1							
2. Familiarity	−0.130^**^	1						
3. Controllability	−0.199^**^	0.462^**^	1					
4. NP	0.356^**^	0.003	−0.031	1				
5. PP	−0.041	0.191^**^	0.208^**^	0.246^**^	1			
6. PH	0.114^**^	0.059^*^	0.060^*^	0.466^**^	449^**^	1		
7. PF	0.310^**^	−0.038	−0.014	0.651^**^	214^**^	535^**^	1	
8. FT	−0.129^**^	0.306^**^	0.291^**^	0.145^**^	590^**^	317^**^	0.086^**^	1
M ± SD	238.3 ± 57.58	22.8 ± 4.06	22.61 ± 3.37	2.9 ± 0.87	3.72 ± 0.67	3.03 ± 0.75	2.80 ± 0.81	2.8 ± 0.81

* *p < 0.05,*

** *p < 0.01.*

*NP, Negative past; PP, Positive past; PH, Present Hedonism; FT, future time; PF, Present Fatalistic*.

**Figure 1:**
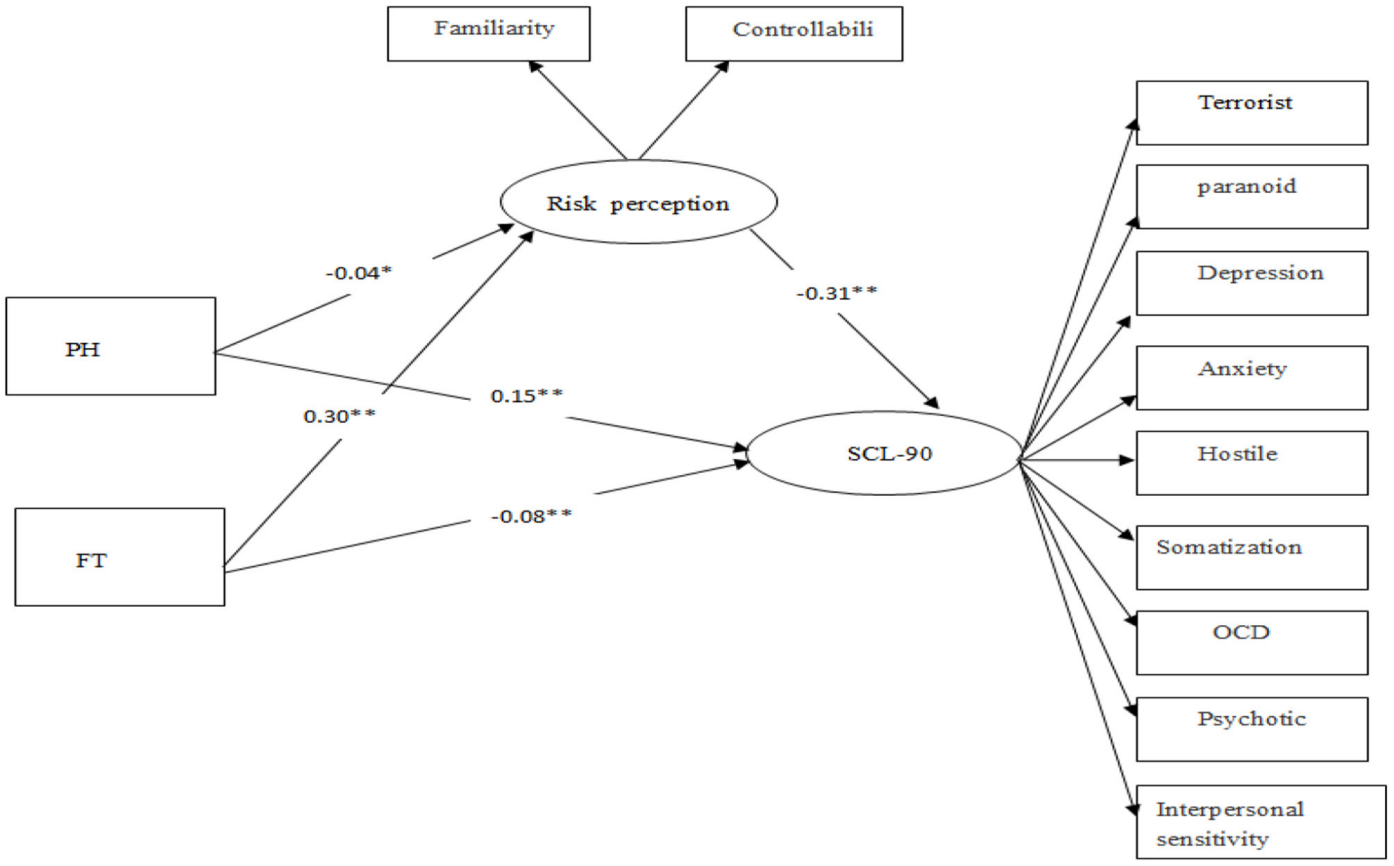
Final model showing moderated mediation; RP mediates TP effect on MH. ^*^p < 0.05, ^**^p < 0.01.

We first examine the relationship between the dimensions of TP, RP, and MH. Through the correlation analysis, we find that the negative past dimension of TP is not related to RP, the positive past dimension is not associated with MH, and the fatalism dimension is not associated with RP, which is different from previous studies (13). One reason for this is that the mental health level of college students is worse during the COVID-19 pandemic than usual. Furthermore, our research also found that most college students have moderate to severe mental health problems. Another reason for this is that the outbreak makes them more concerned about the current situation, while the past positive experience has less buffering effect on the present. Based on these, to confirm an indirect result of TP on MH through the mediating variable of RP, we propose a mediator model in AMOS20.0 (Figure 1).

The results showed that the model fit well (χ^2^*/df* = 13.463, *RMESEA* = 0.1, *IFI* = 0.965, *RFI* = 0.948, *NFI* = 0.963). Figure 1 shows the indirect bootstrap effects for the mediating effect of RP. The results show that when RP is the mediating variable of FT view and MH, the 95% confidence intervals with indirect effect [−0.144, −0.071] and direct effect [−0.144, −0.027] do not contain zero. Thus, RP's moderation effect is confirmed, accounting for 53% of the total effect. When RP is the mediating variable between PH and MH, the 95% confidence intervals with indirect effect [0.0007, 0.031] and direct effect [0.113, 0.205] do not contain zero, which was statistically significant. Thus, RP's moderation effect is confirmed, accounting for 7.6% of the total effect.

## Discussion

This study explored the impact of COVID-19 on the mental health of college students from the perspective of time insight and risk perception. Our research has found that college students generally have psychological health problems during the COVID-19 pandemic. More than 65% of college students have moderate to severe mental health problems. Results of correlation analysis have shown that mental health has a significant positive correlation with the dimension of negative past, the dimension of present-hedonistic, and the dimension of fatalistic view, and has a significant negative correlation with the future-time view. Mediating effect test has found that risk perception plays a part in the mediating effect between the dimension of future time perspective and the mental health of college students. In addition, risk perception has a partial mediating effect between the dimension of present-hedonistic and the mental health of college students.

The high detection rate of mental health problems among college students during COVID-19 is consistent with research results at home and abroad. And research also shows that the closure of schools and colleges harmed more than 91% of students worldwide (21). The COVID-19 quarantine hurts young people's physical and mental health (22). In addition, the delayed beginning of term has led to a lack of a regular learning atmosphere for college students, confusion, boredom, and lack of communication in daily life; all of those will cause college students to have a lower level of mental health (4, 23, 24). This reminds us that the mental health of college students during the COVID-19 pandemic cannot be ignored. Correlation analysis has shown that mental health has a significant positive correlation with the dimension of negative past, the dimension of present-hedonistic, and the dimension of fatalistic view, and has a significant negative correlation with the future-time view. An individual who is full of hope for the future and has a clearer goal has a stronger belief and will to complete their plan. At the same time, they will also have a more positive attitude and way to overcome difficulties (25). Individuals' emotional experience, attitudes, and perceptions for time will affect their own mental health. College students who hold the view of present-hedonistic pay more attention to the feelings of the present and do not have many plans for the future. Therefore, the sudden COVID-19 pandemic has enhanced the negative emotional experience of college students who hold the view of present-hedonistic. They feel the threat of the current environment and think that their future life is gloomy and may disobey the government's measures (home isolation and city lockdown). The lack of a sense of control and security of the present will undoubtedly make individuals have a self-denying attitude and cause psychological problems. Time perception insight has a significant impact on the mental health of college students, especially individuals who hold a view of future time. Their level of mental health is relatively high. This reminds us that whether it is during the COVID-19 pandemic or in daily life, we should all be concerned about the future and have confidence in the future.

During the COVID-19 outbreak, risk perception had a partial mediating effect on the future time perspective dimension, present enjoyment dimension, and college students' mental health. That is, time perception directly affects college students' mental health level and indirectly affects the mental health level of college students through risk perception. The more positive emotional experience, successful experience, self-reflection, and other factors an individual has in the past will affect the individual having more positive memories of the past. It can make the individual more confident, calm, and able to withstand setbacks when facing the current situation. During the COVID-19 epidemic, college students who were full of hope for the future were more likely to believe that COVID-19 was controllable and were more confident in national policies and government measures. As pointed out in previous studies, an important factor affecting risk perception was the perception of government participation attitude and trust (26). When college students perceive the government's commitment to overcoming the outbreak to be high, the more hopeful they are about the future and the more they feel the outbreak is controlled, so their individual psychological health level is higher. When an individual's confidence in the future will produce clear objectives, their behavior will be more positive and active. More and more research has shown that maintaining a hopeful, goal-oriented future direction is good for young people's mental health (5, 23, 24). These changes in our perception of time and our view of the future could significantly impact our health and well-being (27). Abramson, Metalsky, and Alloy proposed hopelessness depression: when adverse events occur, individuals with negative emotions or styles are more likely to make adverse inferences and suffer from depression (28).

In the same way, individuals who enjoy the present are prone to make negative judgments on adverse events such as epidemics because individuals who e pay more attention to the enjoyment of the present make fewer plans for the future. In the face of the sudden COVID pandemic-19, they are prone to feel uneasy and afraid, which will affect their mental health. Therefore, how to intervene in individuals' time attitude and actively conduct epidemic risk expectations becomes key to alleviating personal anxiety and maintaining people's mental health during the epidemic period. The individual emotional response is an essential factor affecting risk perception, and negative emotions play a significant role in risk perception (23). This also explains why risk perception has a mediating effect on future time perspective dimension, negative past dimension, and mental health. When a major public health crisis occurs, the negative subjective perception of the situation is an essential factor leading to a low level of mental health (29). Therefore, attention should be paid to the subjective risk perception of college students because the individual's subjective perception of their risk of infection may not be consistent with the objective situation (24). Since individuals' subjective perception of their risk of infection may be inconsistent with the objective situation, it is necessary to focus on college students' subjective risk perception and guide them to maintain correct and positive subjective perception of COVID-19 risk.

## Conclusion

This study found that during the outbreak of COVID-19, the present hedonism time perspective and future time perspective could affect college students' mental health not only directly but also indirectly through risk perception. It is also recommended to increase people's familiarity with COVID-19 during the COVID-19 outbreak and increase confidence in the controllability of COVID-19 to reduce the impact of the COVID-19 pandemic on people's mental health.

## Deficiencies and Prospects

This research also has some shortcomings; first of all, the basic information of the participants had no further detailed division, including professional subjects, whether participants had close contact with COVID-19 patients, or whether participants were involved in the pandemic control or prevention, which may all have certain influences on college students' psychological health, It is hoped that the experience of this study can be used for reference in future studies. Secondly, this study only sampled college students from provinces other than Hubei, China, and did not compare with college students nationally and those with COVID-19 infection nearby. Finally, the study is a cross sectional study and did not track how college students' mental health changed as the pandemic progressed. Future studies can continue to track the outbreak era dynamic change and other influencing factors of college students' mental health problems.

## Data Availability Statement

The raw data supporting the conclusions of this article will be made available by the authors, without undue reservation.

## Author Contributions

All authors conducted data collection, data processing, article writing, article discussion, and modification.

## Funding

This research was supported by the National Natural Science Foundation of China (Project 31960185), 2021 Northwest Normal University Young Faculty Research Ability Enhancement Program (Project 2946), and Lanzhou Thirteenth Five-Year Plan Educational Science Planning Project (Project LZ2019-0250).

## Conflict of Interest

The authors declare that the research was conducted in the absence of any commercial or financial relationships that could be construed as a potential conflict of interest.

## Publisher's Note

All claims expressed in this article are solely those of the authors and do not necessarily represent those of their affiliated organizations, or those of the publisher, the editors and the reviewers. Any product that may be evaluated in this article, or claim that may be made by its manufacturer, is not guaranteed or endorsed by the publisher.
